# Sonochemical conversion of CO_2_ into hydrocarbons: The Sabatier reaction at ambient conditions

**DOI:** 10.1016/j.ultsonch.2021.105474

**Published:** 2021-02-02

**Authors:** Md Hujjatul Islam, Odne S. Burheim, Jean-Yves Hihn, Bruno.G. Pollet

**Affiliations:** a Hydrogen Energy and Sonochemistry Research Group, Department of Energy and Process Engineering, Norwegian University of Science and Technology (NTNU), Trondheim, Norway; b UTINAM UMR 6213 CNRS, Université Bourgogne Franche-Comté, Besançon, France

**Keywords:** Ultrasound, Sonochemistry, Carbon dioxide, Hydrocarbons, Sabatier reaction, Seawater

## Abstract

In this study, we investigated an alternative method for the chemical CO_2_ reduction reaction in which power ultrasound (488 kHz ultrasonic plate transducer) was applied to CO_2_-saturated (up to 3%) pure water, NaCl and synthetic seawater solutions. Under ultrasonic conditions, the converted CO_2_ products were found to be mainly CH_4_, C_2_H_4_ and C_2_H_6_ including large amount of CO which was subsequently converted into CH_4_. We have found that introducing molecular H_2_ plays a crucial role in the CO_2_ conversion process and that increasing hydrogen concentration increased the yields of hydrocarbons. However, it was observed that at higher hydrogen concentrations, the overall conversion decreased since hydrogen, a diatomic gas, is known to decrease cavitational activity in liquids. It was also found that 1.0 M NaCl solutions saturated with 2% CO_2_ + 98% H_2_ led to maximum hydrocarbon yields (close to 5%) and increasing the salt concentrations further decreased the yield of hydrocarbons due to the combined physical and chemical effects of ultrasound. It was shown that CO_2_ present in a synthetic industrial flue gas (86.74% N_2_, 13% CO_2_, 0.2% O_2_ and 600 ppm of CO) could be converted into hydrocarbons through this method by diluting the flue gas with hydrogen. Moreover, it was observed that in addition to pure water, synthetic seawater can also be used as an ultrasonicating media for the sonochemical process where the presence of NaCl improves the yields of hydrocarbons by ca. 40%. We have also shown that by using low frequency high-power ultrasound in the absence of catalysts, it is possible to carry out the conversion process at ambient conditions i.e., at room temperature and pressure. We are postulating that each cavitation bubble formed during ultrasonication act as a “micro-reactor” where the so-called *Sabatier reaction* -$CO_{2}+4H_{2}\overset{\mathrm{Ultrasonication}}{\to }CH_{4}+2H_{2}O$ - takes place upon collapse of the bubble. We are naming this novel approach as the “Islam-Pollet-Hihn process”.

## Highlights

• “The Islam-Pollet-Hihn process” is presented.
• CO_2_ reduction aided by ultrasound in ambient conditions and without the use of a catalyst.
• A hydrocarbon yield of ca. 5% is achieved when 2% CO_2_ and 98% H_2_ in 1.0 M NaCl solution is sonicated.
• Increasing hydrogen concentration increases the yield of hydrocarbons.
• The CO_2_-methanation reaction may occur either by direct methanation or by radical attack induced by cavitation.

## 1 Introduction

CO_2_ is the major contributor to global climate change. Around 80–90% of the total global CO_2_ emission comes from fossil fuel combustion. This emission has been increasing by 2.7% annually over the past decades [1]. The CO_2_ levels have risen above 400 ppm and it is thought that it will not decrease for many years. The scientific consensus is that these emission levels are unsustainable and must be curbed if mankind is to avoid irreparable damage to global ecosystems [2]. Immense research and investment have been carried out for efficiently capturing CO_2_ and converting it into useful hydrocarbon fuels since the early 21st century [3].

Conversion of CO_2_ into hydrocarbons is of specific interest since this pathway can contribute to minimizing climate change while obtaining valuable products. There are several possible methods for turning CO_2_ into a fuel, including chemical, photochemical, electrochemical (CO2RR - electrochemical CO_2_ reduction reaction) and biochemical methods [3]. Although, most of these methods are energy intensive and less efficient to be economically viable. Industrially, the most widely used method to convert CO_2_ into hydrocarbons, is called the *Sabatier reaction*, also known as the *Sabatier process.*

Sabatier and Senders introduced this reaction first time in the beginning of the 20th century. It was mainly used to remove CO_2_ from the feed gas from ammonia synthesis. Recently hydrogen (H_2_) has gained renewed interest in the field of power-to-gas (P2G) technology. According to the Sabatier reaction, one mole of CO_2_ reacts with four moles of H_2_ to produce one mole of methane. This synthetic route is renewable and sustainable if the required hydrogen is produced via water electrolysis using renewable electricity such as hydro, wind or solar power. This is an exothermic reaction and the stoichiometry is shown in equation (1) [4].

(1) $$CO_{2}+4H_{2}↔CH_{4}+2H_{2}O\;\Delta H_{298}^{0}=-165\;\text{kJ}/\text{mol}$$

This strongly exothermic reaction is accompanied by a mildly endothermic reverse water–gas shift reaction (2) and an exothermic CO methanation (2) [4], [5], [6].

(2) $$CO_{2}+H_{2}↔CO+H_{2}O\;\Delta H_{298}^{0}=+41\;\text{kJ}/\text{mol}$$

(3) $$3H_{2}+CO↔CH_{4}+H_{2}O\;\Delta H_{298}^{0}=-206\;\text{kJ}/\text{mol}$$

The overall process is very exothermic, and the reaction is favored at lower temperatures. However, at low temperatures, the reaction kinetics are poor, and a catalyst needs to be used to overcome the kinetic limitations. Different catalysts have been employed for the methanation reaction such as, Ni, Ru and Rh. Nickel (Ni) is the most widely used catalyst due to its high selectivity towards methane and its low cost. The operating temperature for Ni-based catalyst are usually kept below 550 °C in order to prevent catalyst deactivation.

However, the Sabatier process is an energy intensive process. In order to overcome this energy dependency on CO_2_ fixation, many other processes have evolved over the last few decades. These processes are mainly the electrochemical CO_2_ conversion [7], photocatalytic conversion [8], modified Fischer-Tropsch (FT) [9] and the biochemical routes [10], [11]. All these methods have advantages and disadvantages and depend upon the nature of the CO_2_ input, that is, its purity and temperature. In the search of an energy efficient CO_2_ conversion process, we have investigated the possibility to use power ultrasound to convert CO_2_ into hydrocarbons at ambient conditions i.e., at room temperature and pressure and without using any catalytic materials.

It is well-known that when a liquid, such as water, is subjected to ultrasound in the range of 20 kHz to 1 MHz, microscopic bubbles also known as cavitation bubbles are formed. Cavitation bubble collapse leads to near adiabatic heating of the vapour that is trapped inside the bubble creating the so-called “hotspot” in the fluid where high temperatures (ca., 5,000 K) and high pressure (ca. 2,000 atm) are generated. At these extreme conditions, water vapour is ‘pyrolyzed’ into hydrogen (H^•^) and hydroxyl radicals (OH^•^) known as water sonolysis. However, it was observed that the production of radicals is ultrasonic frequency dependent and it was found that the yield of radicals are maximum in the range 340–500 kHz [12]. We speculate that the millions of cavitation bubbles produced by ultrasonication may act as micro-reactors where *Sabatier reaction* can take place in the presence of hydrogen which are produced during the sonolysis of water or supplying excess hydrogen into the system via water electrolysis [13]. In this study, we present the proof of this concept through rigorous experimental procedures addressing the key parameters that govern the *Sabatier reactions* at ambient conditions under power ultrasound. We have named this novel approach as the “Islam-Pollet-Hihn process” which is an ultrasound-assisted *Sabatier process* at ambient conditions and in absence of a catalyst.

## 2 Experimental

### 2.1 Experimental reactor setup

**Fig. 1:**
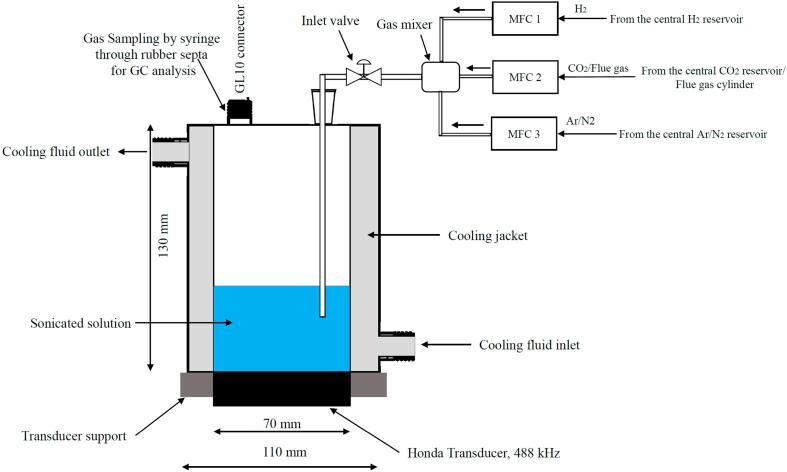
Schematic illustration of the experimental setup. Here, MFC = Mass Flow Controller, GC = Gas Chromatograph.

The CO_2_ conversion experiments were performed using a 488 kHz ultrasonic transducer of 70 mm diameter manufactured by Honda Electronics Co., LTD. The ultrasound emitting surface area is approximately of 1.54 cm^2^. This transducer is fitted to a specially designed glass reactor of 523 ml volume. The reactor has an inner diameter of 70 mm which is equal to the transducer diameter. The outer diameter of the reactor is 110 mm. The outer space is used as the cooling jacket in order to ensure efficient cooling. The reactor is then clamped with the transducer support. A silicon sheet of 0.5 mm thickness is placed in between the glass reactor and transducer support in order to ensure complete sealing (Fig. 1).

The inner vessel of the reactor has two ports. One port equipped with an NS14 glass joint which is used to insert a glass tube inside the reactor for gas bubbling. Another port is equipped with a GL10 thread. A screw cap with rubber septa is used to close this port. Gas samples for Gas Chromatography (GC) analysis were collected through the rubber septa using a Hamilton gas tight syringe (1000 series, 1 ml inner volume) equipped with SampleLock feature.

Three Mass Flow Controllers (MFC) from Alicat Scientific were used for mixing the gases in desired composition. The inlet of the MFCs were connected with the central gas reservoir or gas cylinders such as flue gas/calibration gas. The outlet of the each MFCs were connected with a gas mixture in order to ensure efficient mixing of the desired gases before entering into the reactor. The output pressure of the MFCs were set to 1,100 mbar which was also equal to the reactor pressure. A gate valve was placed in between the gas mixture and the inlet of the reactor in order to ensure air tightness inside the reactor.

For the sono-CO_2_ conversion experiments, ultrapure water (18.2 MΩ), NaCl (ACS reagent ≥ 99.0%, Sigma Aldrich) solution of different concentrations and synthetic seawater was used as ultrasonication media. The synthetic seawater was prepared according to the chemical components reported by Kester *et al.* [14] which has a salinity of 35. The components of the synthetic seawater are presented in Table 1. Synthetic flue gas was purchased from Linde which was composed of 86.74% N_2_, 13% CO_2_, 0.2% O_2_ and 600 ppm of CO.

**Table 1:** List of chemical components in synthetic seawater for a salinity of 35.

Salts	Concentration (g/L)	Molar concentration (M)
NaCl	23.93	0.4096
MgCl_2_	5.079	0.0249
Na_2_SO_4_	3.994	0.0281
CaCl_2_	1.123	0.0101
KCl	0.667	0.0089
KBr	0.098	0.00082
H_3_BO_3_	0.027	0.00044
SrCl_2_	0.024	0.00009
NaF	0.003	0.00007
NaHCO_3_	0.196	0.00233

At first, 200 ml of solution was transferred into the reactor and then desired gas compositions were bubbled into the water for 30 min by keeping the outlet port (GL10 threaded) marginally open. After 30 min, the outlet port was completely closed. As soon as the reactor pressure had reached 1,100 mbar, the inlet valve was also closed ensuring a complete airtight system. After that, the ultrasonication started and lasted for 1 h. After 1 h of ultrasonication, gas samples were collected and injected into the GC for analysis. The liquid samples were also collected and analyzed by High Performance Liquid Chromatography (HPLC).

### 2.2 Dosimetry and calorimetry study

The “Weissler dosimetry” (potassium iodide – KI dosimetry) was performed at 5 °C for the ultrasonic frequencies of 20, 210, 326, 408 and 488 kHz according to the method explained by Iida *et al*. [15]. At 488 kHz frequency, the Weissler dosimetry was performed at four different gas saturations such as CO_2_, H_2_, N_2_ and Ar. 200 ml of 0.10 M KI were ultrasonicated for 10 min. Prior to ultrasonication, the solution was bubbled for 10 min with the respective gas. After 10 min of ultrasonication, aliquots of 1 ml were collected and analyzed using a UV–vis spectrophotometer (GENESYS 30, Thermo Scientific).

In order to calculate the Sonochemical Efficiency (SE), acoustic powers (*P*_acoustic_) were determined by the calorimetric method, according to Mason *et al.* [16] and Contamine *et al.* [17]. SE [μmol/kJ] was calculated according to equation (4) [15].

(4) $$\mathrm{SE}=\frac{\mathrm{CV}}{P_{\mathrm{acoustic}}t}$$

Here, *C* [μM] is the concentration of I_3_^-^ , *V* [L] is the solution volume, *P*_acoustic_ [kW] is the acoustic power and *t* [s] is the ultrasonication time.

### 2.3 CO_2_ conversion product analysis

The gaseous products were analyzed using an SRI GC (Model 8610C). The GC was equipped with 3 Hayesep D Packed columns (8600-PKDB 6′ x 1/8″ S.S) with a total length of 18 feet connected in series. Both FID (Flame Ionization Detector) and TCD (Thermal Conductivity Detector) detectors were used to identify and quantify all the gases. The FID was used mainly for analyzing the hydrocarbons such as CH_4_, C_2_H_4_, and C_2_H_6_ and the sensitivity of the detector was set to “HIGH”. The TCD detector was used for analyzing the H_2_, O_2_, N_2_, CO, and CO_2_ . Argon (Ar) was used as carrier gas in the GC. GC was calibrated in a three-point calibration. Different calibration gas mixtures were prepared using the MFCs and injected into the GC for constructing the calibration curve. Then the reaction samples were analyzed against the performed calibration curve. Before analyzing the unknown reaction samples, a known concentration of gas was injected each time in order to check the accuracy of the analysis.

The liquid samples were analyzed using a Shimadzu Prominence *i* series compact HPLC (LC-2030C 3D Plus). The HPLC was equipped with a Shodex SUGAR SH1011 column including two detectors. The detectors were a PDA (Photodiode Array) and a RID (Refractive Index Detector). The HPLC analysis was performed in an isocratic method with the mobile phase (5 mM H_2_SO_4_) at a flowrate of 0.8 ml/min. For calibration of the HPLC, a stock mixture solution made of 0.05 M of ethanol, methanol, formic acid and acetic acid was prepared. Two more samples of 0.01 M and 0.025 M were prepared by diluting the 0.05 M stock solution. The three known concentration samples were then analyzed for constructing the three-point calibration graph. The unknown reaction samples were then analyzed against the calibration curve.

### 2.4 Carbon-based conversion and yield calculations

To study the sonochemical CO_2_ conversion, the overall carbon-based conversion efficiency and yield of CO_2_ converted products were used as figures of merit according to the equations (5) and (6). Carbon-based overall conversion efficiency is the amount of initial carbon in the form of CO_2_ (both gaseous and dissolved in the solution) that is converted into products after 1 h of ultrasonication. Carbon-based yield of products is the amount of carbon present in the product from the initial amount of the total carbon.

Carbon-based overall conversion efficiency:

(5) $$X_{CO_{2}}=\frac{{\left(m_{CO_{2(g)}}+m_{CO_{2(dissolved)}}\right)}_{t=0}-{\left(m_{CO_{2(g)}}+m_{CO_{2(dissolved)}}\right)}_{t=t}}{{\left(m_{CO_{2(g)}}+m_{CO_{2(dissolved)}}\right)}_{t=0}}\times 100$$

(6) $$\mathrm{Carbon}-based\;yield:Y_{i}=\frac{{\left(m_{i(gas)}+m_{i(dissolved)}\right)}_{t=t}}{{\left(m_{CO_{2(g)}}+m_{CO_{2(dissolved)}}\right)}_{t=0}}\times 100$$

Here,

*m*_CO2(g)_ = mass of carbon in gaseous CO_2_.

*m*_CO2(dissolved)_ = mass of carbon in dissolved CO_2_.

*m*_i(gas)_ = mass of carbon in the product *i* in gaseous state.

*m*_i(dissolved)_ = mass of carbon in the product *i* in dissolved state.

The gas concentration in the gaseous state was measured using the gas chromatograms. The amount of dissolved gases was estimated using the Van’t Hoff equation (7) and Henry’s law (8) [18].

(7) $$H\left(T\right)=H_{\mathrm{ref}}\times e^{\left(-K\left(\frac{1}{T}-\frac{1}{T_{\mathrm{ref}}}\right)\right)}$$

(8) $$c_{i}=\frac{p_{i}}{H(T)}$$

Here,

*H*(*T*) = Henry constant at temperature *T*

*H*_ref_ = Henry constant at reference temperature (at STP)

$K=-\frac{\Delta H_{\mathrm{sol}}}{R}$ = constant

*C*_i_ = Molar concentration of the dissolved gas *i*

*p_i_* = Partial pressure of the dissolved gas *i*

After 1 h of ultrasonication, the liquid samples were also collected and analyzed by HPLC. A trace amount of ethanol was found in the liquid samples through the HPLC analysis. However, since the quantity is very small, it was not taken into the consideration when the overall conversion efficiency and yield of different products were calculated.

## 3 Results and discussion

**Fig. 2:**
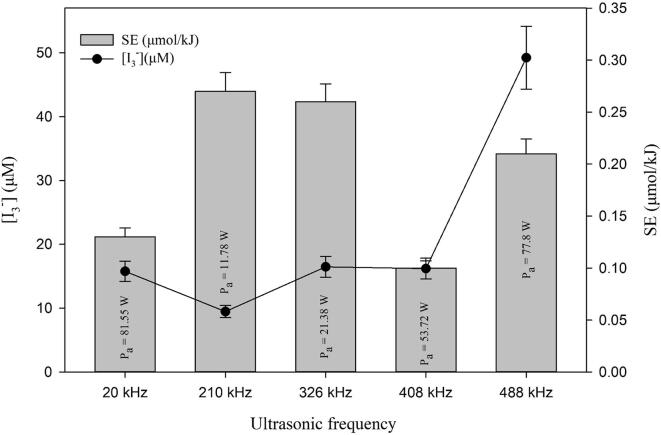
Effect of ultrasonic frequency on the sonochemical activity.

In order to choose the right frequency for the sonochemical CO_2_ conversion experiment, at first the sonochemical activity of the ultrasonic transducers of different frequencies were studied. The energy-specific yield of radicals due to ultrasonication at different frequency is shown in Fig. 2. The transmitted acoustic power (*P*_acoustic_) at 20 kHz (50% amplitude) was found to be the maximum (81.55 ± 0.62 W). The lowest acoustic power (11.78 ± 1.15 W) was found at 210 kHz, and according to equation (4), sonochemical efficiency was maximum at that frequency. On the other hand, the triiodide concentration was found to be maximum at 488 kHz (49.23 μM). This value was ca. three times higher than the value obtained from the 20, 326 and 408 kHz ultrasonic transducers. In addition, the triiodide concentration was five times higher at 488 kHz than at 210 kHz. At this stage of this study, the main focus was to find a sonochemical system with yielded the highest cavitational activity instead of the highest SE. Therefore, the 488 kHz transducer was chosen for all sonochemical CO_2_ conversion experiments.

**Fig. 3:**
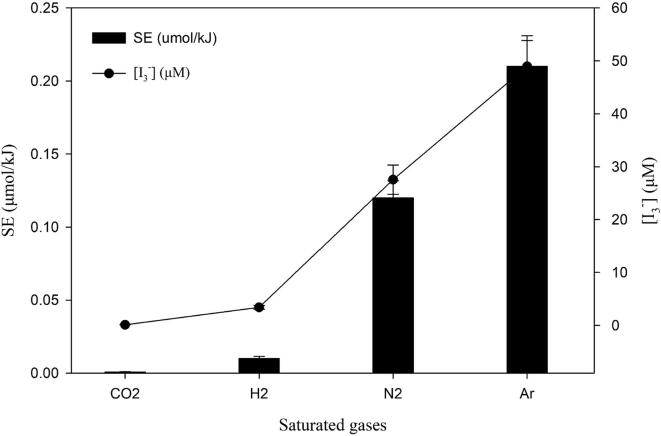
Effect of dissolved gases on the sonochemical activity (488 kHz).

In addition, the cavitational activity in both diatomic and monoatomic gases at 488 kHz was studied and the results are presented in Fig. 3. It was found that the monoatomic gases such as argon (Ar) exhibited the maximum sonochemical efficiency due to its higher polytropic ratio (*γ* = 1.66) and lower thermal conductivities (*λ* = 0.018 W/m.K) compared to N_2_ (*γ* = 1.40, *λ* = 0.024 W/m.K) and H_2_ (*γ* = 1.405, *λ* = 0.0167 W/m.K) [13]. However, hydrogen plays a unique role in sonochemical CO_2_ conversion which is further explained in *section 3.1*. On the other hand, cavitational activity in the presence of dissolved CO_2_ is suppressed almost entirely. Therefore, sonochemical reduction of CO_2_ can be carried out only by mixing with other gases such as Ar, N_2_ or H_2_.

### 3.1 Effect of hydrogen gas concentration

**Fig. 4:**
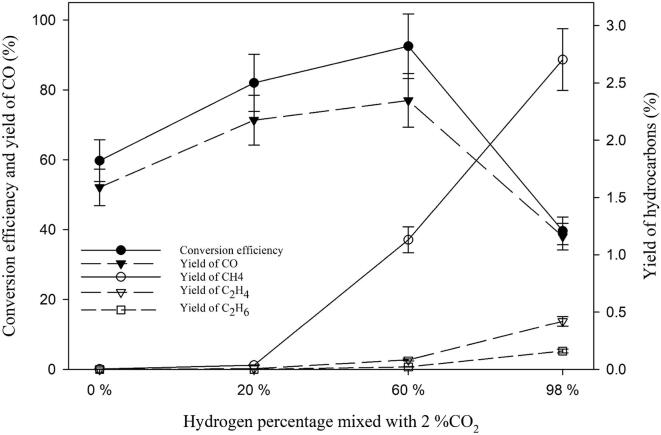
Effect of molecular hydrogen gas concentration on the sonochemical CO_2_ conversion at 5 °C in pure water.

Since, in CO_2_-saturated solutions, cavitation activity is quenched almost entirely, a mixture of CO_2_ with Ar and H_2_ was chosen for the sono-CO_2_ conversion experiments. In order to understand the mechanism of the sono-CO_2_ conversion process, 2% CO_2_ was mixed with three different H_2_ concentrations and ultrasonicated for one hour using pure water as ultrasonicating media at 5 ˚C. In the first set of experiments, no hydrogen (0%) was used but 2% CO_2_ was mixed with 98% Ar. In the second set of experiments, 2% CO_2_ was mixed with 20% H_2_ and 78% Ar. In the third set of experiments, 2% CO_2_ was mixed with 60% H_2_ and 38% Ar and the last set of experiments was performed with 2% CO_2_ and 98% H_2_. The experimental findings are presented in Fig. 4. It was observed that the conversion efficiency increased with increasing hydrogen concentration from 0 to 60%. However, the conversion efficiency drastically decreased when the hydrogen concentration was 98%. It was found that the main sono-CO_2_ reduced product was CO which also followed the same trend as the conversion efficiency.

On the other hand, the yield of hydrocarbons such as CH_4_, C_2_H_4_, and C_2_H_6_ increased with increasing hydrogen concentration. A CH_4_ yield of 2.7% was observed when a mixture of 2% CO_2_ and 98% H_2_ was ultrasonicated at 5 °C. When no hydrogen (0%) was used (2% CO_2_ + 98% Ar), only a trace amount (0.003% yield) of methane was observed. It is possible that during bubble collapse, *in-situ* produced hydrogen through water sonolysis [13], reacts with CO_2_ producing CH_4_ according to the *Sabatier reaction*. When 20% H_2_ is added and 20% Ar is reduced, the yield of CH_4_ was found to be only 0.03%. The ratio between CO_2_ and H_2_ was found to be 1:10 which is larger than the *Sabatier reaction* ratio (1:4). However, when only hydrogen is used with 2% CO_2_, CH_4_ yield increased drastically. Therefore, hydrogen works not only as a hydrogen donor to fulfill the Sabatier ratio, but it also acts as a reducing agent.

Gutierrez *et al*. [19] studied for the first time the effect of hydrogen atom, *H*, in the sonolysis of aqueous solution. They observed that under argon atmosphere, the primary step in the sonolysis of water follows reaction (9).

(9) $$H_{2}O\to H^{\cdot }+OH^{\cdot }$$

However, when hydrogen is present in the system, the hydroxyl radicals (OH•) are scavenged by hydrogen leaving the H^•^ agent free according to the reaction (10)

(10) $$OH^{\cdot }+H_{2}\to H_{2}O+H^{\cdot }$$

OH^•^ is an oxidizing agent whereas H^•^ is a reducing agent. During ultrasonication in the hydrogen atmosphere, the continuous removal of OH^•^ creates an overall reducing environment in the system. Recently, Islam *et al.* [20] postulated that the extreme conditions caused by the cavitation bubble collapse may trigger the homolytic fission of H_2_ molecule producing higher amount of H^•^.

(11) $$H_{2}\overset{\mathrm{Ultrasonication}}{\to }H^{\cdot }+H^{\cdot }$$

Due to the creation of this reducing environment, CO_2_ reduction is facilitated producing more reduced products such as carbon monoxide and hydrocarbons. From Fig. 4, we observe that there is a maximum present in the reduction of CO_2_ to CO at around 50% H_2_. With increasing H_2_, more OH^•^ radicals are scavenged by hydrogen that would re-oxidize the reduced products such as CO and hydrocarbons formed by H^•^ attack. The increase in gas content within the liquid leads to a lower cavitation threshold and intensity of the shock wave released on the collapse of the bubble. It has been observed that the use of monoatomic gases (e.g., He, Ar, Ne) provides more effective cavitation than diatomic gases (e.g., N_2_, O_2_, air). However, molecular hydrogen is a diatomic gas. Increasing the concentration of a diatomic gas usually decreases the overall cavitation activity in the system due to adiabatic compression during bubble collapse. We can observe this phenomenon from the dosimetry study presented in Fig. 3. Due to these two-opposing effects, we may see a maximum point on the conversion of CO_2_ and the yield of CO in Fig. 4. On the other hand, the yield trends of hydrocarbon have an opposite behavior whereby rising H_2_ increases gradually the yields of hydrocarbons. Two possible reasons for this behaviour can be addressed as follows. One reason is the higher amount of available H^•^ with increasing hydrogen concentration. Another reason is the lack of OH^•^ which could re-oxidize hydrocarbons back to CO_2_. Therefore, if one wants to convert CO_2_ into hydrocarbons, then higher hydrogen concentration is the optimal option. If one wants to reduce CO_2_ into CO, then an equal mixture of Ar and H_2_ would provide the maximum yield.

### 3.2 Effect of CO_2_ concentration

**Fig. 5:**
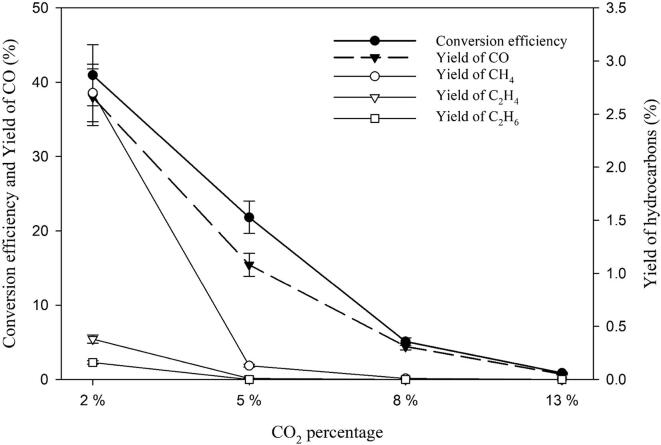
Effect of CO_2_ concentration on the sonochemical conversion of CO_2_ at 5 °C in pure water.

The effect of CO_2_ concentration on the sono-CO_2_ reduction was studied and the results are presented in Fig. 5. In this set of experiments, 2%, 5%, 8% and 13% CO_2_ were mixed with 98%, 95%, 98% and 87% H_2_ and was ultrasonicated for 1 h at 5 °C using pure water as sonicating media. It was found that increasing CO_2_ concentrations decreased CO_2_ conversion efficiency and CO yield, and the yields of the hydrocarbons also gradually decreased. For example, increasing the CO_2_ concentration from 2% to 5% decreased the yield of CH_4_ from 2.7% to 0.13%. At 13% CO_2_ concentration, a very trace amount (8 × 10^-4^%) of CH_4_ yield was observed. Conversion efficiencies also decreased from 41% to 0.88% when CO_2_ concentration increased from 2% to 13%.

These findings suggest that CO_2_ concentration has an effect on the cavitational activity. Even the presence of 13% CO_2_ can almost completely quench the acoustic activity in the system. Dosimetry study (Fig. 3) also revealed a similar observation where very negligible values of sonochemical efficiency was obtained when 0.10 M KI solution was ultrasonicated. These findings are in very good agreement with those obtained by Merouani *et al.* [21] and Kerboua *et al.* [22] who studied the mechanism of pure CO_2_-quenching sonochemical processes through numerical method. They claimed that CO_2_ may reduce or even suppress the yield of OH radicals from a single acoustic bubble. This is mainly due to the very high solubility of CO_2_ (46-fold higher than air) in the solution compared to other traditional gases used in sonochemistry. Due to its high solubility, bubble–bubble coalescence occurs more in the presence of CO_2_ than other gases, as well as the presence of these large bubbles reduces drastically the cavitational activity. Thus, CO_2_-saturation may lead to total disappearance of chemical activity. Therefore, in order to avoid bubble–bubble coalescence, a low concentration of CO_2_ is beneficial for carrying out any sonochemical effects. According to Fig. 5, a CO_2_ concentration less than 3% is “ideal” for conversion of CO_2_ into hydrocarbons.

### 3.3 Effect of temperature

**Fig. 6:**
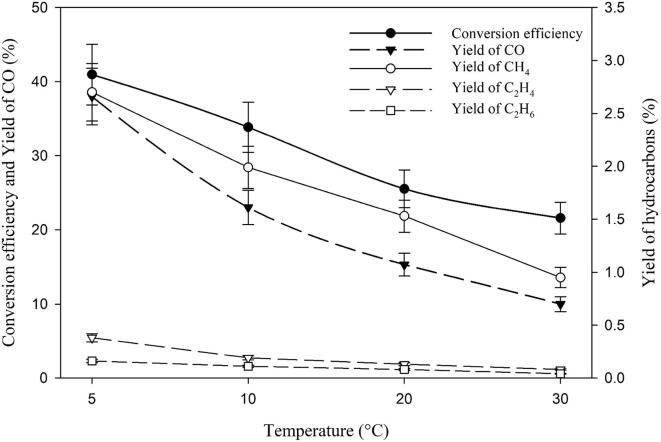
Effect of temperature on the sonochemical CO_2_ conversion in pure water with a gas concentration of 2% CO_2_ and 98% H_2_.

2% CO_2_ mixed with 98% H_2_ in water was ultrasonicated for 1 h at temperatures of 5, 10, 20 and 30 °C and the conversion efficiencies, CO yields and hydrocarbon yields were generated as shown in Fig. 6. It can be observed that increasing temperature decreases the conversion efficiency, yields of CO and hydrocarbons. Almost a 50% decrease in the methane yield is observed by just increasing the temperature from 5 °C to 10 °C. These findings suggest that CO_2_ conversion to hydrocarbons is favorable at low temperatures. A temperature ranges from 2 to 5 °C is advantageous since operating below these temperatures has the risk of freezing the solution when pure water is used, for example.

The reason for the deterioration of the sono-CO_2_ process with increasing temperature can be attributed to the basic principle of sonochemistry in pure water. Increasing temperature decreases the polytropic index (*γ*) of gases, and when the liquid temperature increases, it causes less violent collapse of the cavitation bubble due to the decrease of the polytropic index. Less violent collapse leads to lower internal bubble temperatures. Lower internal bubble temperature lowers the formation of free radicals by the decomposition of water i.e. sonolysis [13]. In addition, quantity of water vapour trapped inside the bubble increases with increasing temperature. It is also known that increasing temperature quenches the cavitation process. Therefore, increasing temperature decreases the global cavitational activity of the system leading to the decrease in the sono-CO_2_ conversion efficiency. In other words, temperature has a significant effect on the sono-CO_2_ conversion process.

### 3.4 Effect of hydrogen on the CO_2_ conversion from flue gas

**Fig. 7:**
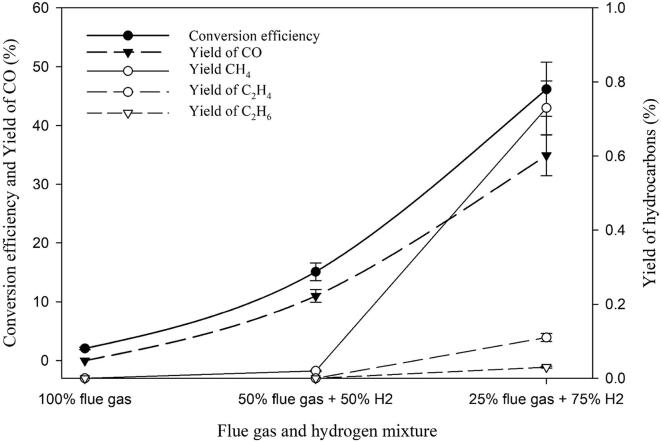
Effect of molecular hydrogen gas concentration on the sonochemical CO_2_ conversion process in the flue gas at 5˚C in pure water.

Conversion of flue gas into hydrocarbon fuels is a specific interest since this process can significantly reduce the CO_2_ emission into the atmosphere while producing valuable fuels. The possibility of converting flue gas into hydrocarbons through the sonochemical method was investigated. The main constituent of a typical flue gas from a coal-fired power plant is: 87% N_2_ along with 13% CO_2_ and trace amount of CO and O_2_. From the initial study on the effect of CO_2_ concentration on the sono-CO_2_ process presented in *section 3.2* (Fig. 5), it was found that the CO_2_ conversion efficiency was very negligible (0.88%) at 13% CO_2_ concentration. Therefore, using ultrasound directly on water-based solutions saturated with flue gas is not a promising strategy. Investigation was performed by mixing the flue gas with H_2_ at two different concentrations (50% flue gas + 50% H_2_, 25% flue gas + 75% H_2_) and the results are shown in Fig. 7.

When a solution made of 100% flue gas in pure water was irradiated with ultrasound at 5 °C, only a 2% conversion efficiency was obtained with a methane yield of 9 × 10^-4^%. Mixing with hydrogen increases the conversion and yield significantly. When 50% flue gas was mixed with 50% H_2_, conversion efficiency was found to be 15% with a methane yield of 0.015%. Diluting the flue gas with more hydrogen (25% flue gas + 75% H_2_) increases both the conversion efficiency and yields of products. A conversion efficiency of ca. 46% was observed with a methane yield of 0.72%. In addition, hydrocarbon with higher carbon numbers such as C_2_H_4_ and C_2_H_6_ were also observed with increasing the hydrogen concentration. When the flue gas was diluted with 75% H_2_ , the CO_2_ concentration in the mixed gas dropped from 13% to 3% which was close to the threshold maximum limit of a meaningful sono-CO_2_ conversion process. The yield of methane from diluted flue gas was still lower when compared to our reference point (2% CO_2_ + 98% H_2_). This interesting finding could be due to the presence of an additional diatomic gas (N_2_ - >80%) which lowered the global cavitational activity. In other words, and from our conditions, CO_2_ conversion using ultrasound from 100% flue gas in water is not feasible. However, mixing the flue gas with H_2_ to maintain the CO_2_ concentration lower than the threshold concentration (3%) increases the CO_2_ conversion efficiency and yield of hydrocarbons significantly.

### 3.5 Effect of NaCl concentration and synthetic seawater

**Fig. 8:**
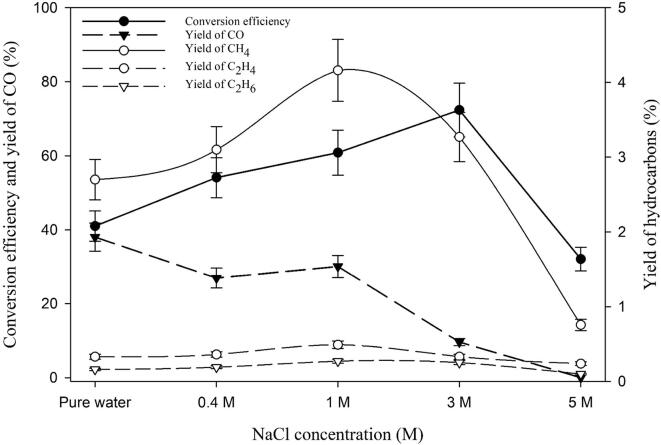
Effect of NaCl concentration on the sonochemical CO_2_ conversion process in a gas mixture of 2 %CO_2_ and 98 %H_2_ at 5 °C.

The effect of NaCl concentration on the sono-CO_2_ process was investigated using 2% CO_2_ and 98% H_2_ gas mixture at 5 °C. Various NaCl concentrations (0.40 M, 1.00 M, 3.00 M and 5.00 M) were used along with pure water as “reference” and the results are presented in Fig. 8. NaCl concentrations have a complex effect on the sono-CO_2_ process. It may be observed that the conversion efficiency increased with increasing NaCl concentration up to 3.00 M and then drastically decreased at 5.00 M. However, the yields of hydrocarbons showed a different trend whereby the yield increased up to 1.00 M and then started decreasing with increasing salt concentration. At 1.00 M NaCl concentration, the yield of methane had a maximum at around 4.2%. These observations can be explained through the study by Pflieger *et al.* [23] where they studied the effect of NaCl concentration on the sonochemistry and sonoluminescence in aqueous solutions. It was shown that the NaCl concentration has multiple effects on the sonochemistry of aqueous solution. For example, they found that the yields of H_2_ and H_2_O_2_ decreased with increasing NaCl concentration due to the combined physical and chemical effects of ultrasound. Increasing NaCl concentration decreased the solubility of gases and increasing the viscosity of the solution. The combined effects of this leads to the changes in the amount of inertial cavitation bubbles. Thus, the global active bubble population decreases due to the decreasing gas solubility. On the other hand, under ultrasonication, new radicals such as Na^•^ and Cl^•^ are formed which react with hydroxyl radicals to form new chemical species such as sodium hydroxide (NaOH). In addition, the effect of salt concentration also depends upon the nature of dissolved gases. As an example, under helium (He) atmosphere, the solution is more acidic due to the formation of H^+^, whereas under Ar atmosphere, the solution is more alkaline i.e., producing NaOH.

**Fig. 9:**
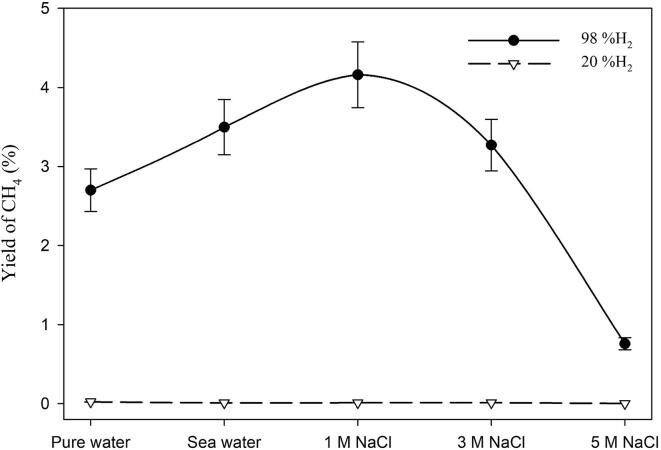
Combined effect of molecular hydrogen concentration and NaCl concentration on the CH_4_ yield from 2% CO_2_ at 5 °C.

**Fig. 10:**
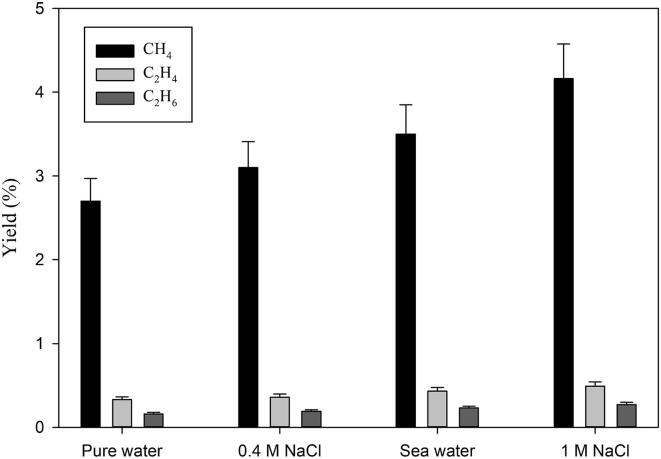
Effect of the analyte on the hydrocarbon yield from 2% CO_2_ − 98% H_2_ at 5 °C.

**Fig. 11:**
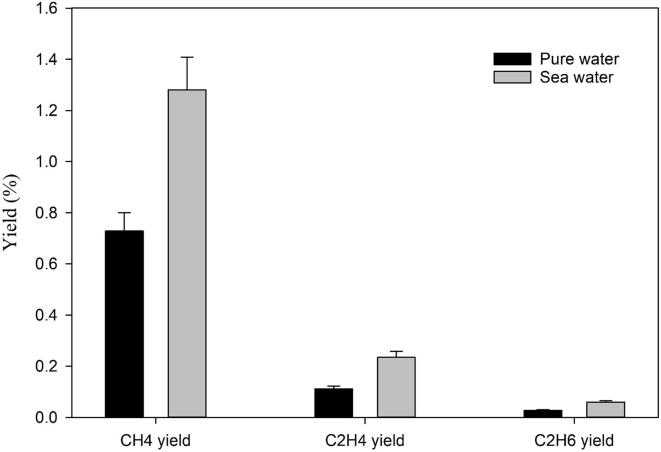
Comparison between synthetic sea and pure waters in hydrocarbon yield from Flue gas (25% flue gas + 75% H_2_) at 5 °C.

In our conditions, the CO_2_ conversion experiments were performed under hydrogen atmosphere. H_2_ has a different role in CO_2_ conversion where it acts as a reducing agent in addition to the hydrogen donor for the Sabatier ratio CO_2_:H_2_ = 1:4. Hydrogen molecules scavenge the hydroxyl radicals and thus create a reducing environment in the system which is prominent until 1.00 M of the NaCl concentration is used. This phenomenon is clearer from Fig. 9 where the effect of hydrogen concentration and NaCl concentration clearly affects the methane yield. When 2% CO_2_ is mixed with 20% H_2_, the yield of methane is not affected by the NaCl concentration at all. However, when 2% CO_2_ is mixed with 98% H_2_, the methane yield increases up to 1.00 M NaCl concentration and then starts decreasing until 5.00 M. At 1.00 M NaCl, an optimal condition exists where there is a balance between the global population of inertial cavitation bubbles and the amount of hydrogen peroxide (H_2_O_2_) formation by hydroxyl radical recombination. Further increase of the salt concentration has a detrimental effect on the sono-CO_2_ conversion where physical effect (increase in viscosity and decrease in gas solubility) is predominant. Under these conditions, the amount of cavitation bubbles is so low that even high concentrations of hydrogen are not enough to overcome this negative effect. Experiments were also performed in synthetic seawater with 2% CO_2_ mixed with 20% and 98% H_2_ respectively. The salinity of the seawater was 35 g/L (0.60 M). The trend of methane yield in synthetic seawater follows the regular NaCl concentration pattern as seen in Fig. 9, Fig. 10. Although in seawater, there are 10 different chemical compounds present, it appears that the different chemicals do not have any additional effects. This is even clearer from Fig. 10. The yield of all the hydrocarbons gradually increases from pure water to 1.00 M NaCl. The molarity of NaCl in seawater is 0.40 M and the total salt concentration in synthetic seawater is 0.60 M. This might be the reason why seawater gives higher yields than 0.40 M NaCl. In addition, the effect of seawater on the sono-CO_2_ conversion process from diluted flue gas (25% flue gas + 75% H_2_) was also studied and it is presented in Fig. 11. As expected, the yield of hydrocarbons in seawater increases significantly (40% increase) compared to pure water. This finding indicates that the CO_2_ content of the industrial flue gas can be efficiently converted into hydrocarbon fuels by using seawater as ultrasonication media and diluting the gas with H_2_.

**Fig. 12:**
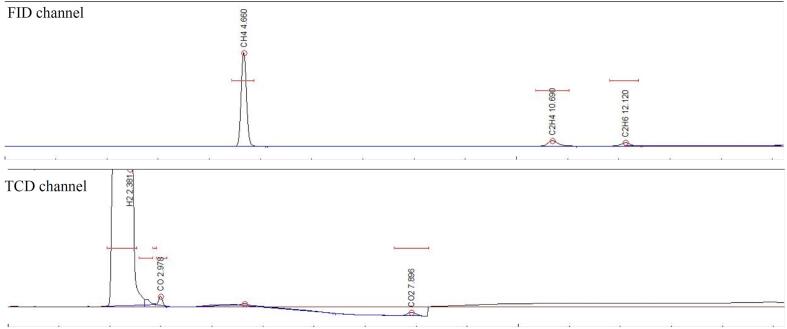
Gas Chromatogram (GC) of 2% CO_2_ + 98% H_2_ in 1.00 M NaCl solution.

**Fig. 13:**
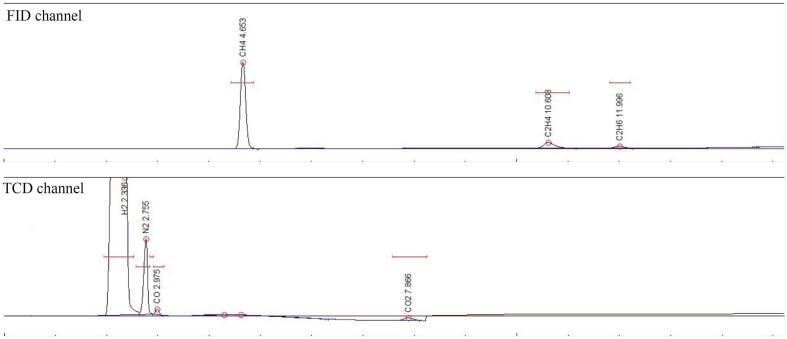
Gas Chromatogram (GC) of 25% flue gas + 75% H_2_ in pure water.

The gas chromatograms obtained from the GC analysis after 1 h of ultrasonication is presented in Fig. 12, Fig. 13. GC analysis was also performed every time before the sonication and no hydrocarbons were detected. Fig. 12 shows the gas chromatogram of 2% CO_2_ + 98% H_2_ in 1.00 M NaCl solution at 5 °C after 1 h of ultrasonication. The hydrocarbon (CH_4_, C_2_H_4_ and C_2_H_6_) peaks are visible in the FID channel whereas the H_2_, CO and CO_2_ peaks are visible in the TCD channel. Fig. 13 shows the gas chromatogram of 25% flue gas and 75% H_2_ in pure water at 5 °C after 1 h of ultrasonication. The N_2_ gas present in the flue gas is visible in the TCD channel.

## 4 Mechanisms

The *Sabatier process* at ambient conditions is a novel process and to the best of our knowledge, this is the only study on the ambient conditions *Sabatier process* using ultrasound. Therefore, the explicit mechanism(s) of the process is still unknown. However, from our findings and those found by the early works performed by Henglein *et al.* [24] and Harada *et al.* [25], we have attempted to provide possible and conceivable mechanisms of the process.

Mechanism 1: Ultrasound induced direct CO_2_ methanation

The *Sabatier reaction* is the combination of the reverse water gas shift reaction (Equation (2)) and CO methanation (Equation (3)). The extreme conditions formed during the cavitation bubble collapse can directly decompose or deoxidize CO_2_ into CO according to the equation (11).

(12) $$CO_{2}\overset{\mathrm{Ultrasonication}}{\to }CO+O$$

Then, the carbon monoxide gas undergoes the methanation process according to reaction (13).

(13) $$3H_{2}+CO\overset{\mathrm{Ultrasonication}}{\to }CH_{4}+H_{2}O$$

**Fig. 14:**
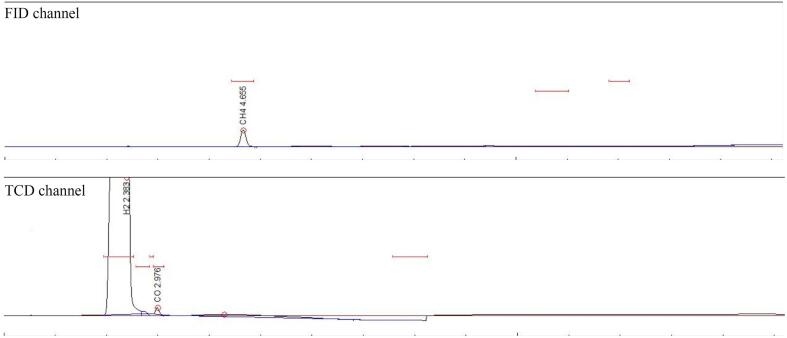
Gas Chromatogram (GC) of 2% CO + 98% H_2_ in pure water.

Experiments were also carried out using 2% CO mixed with 98% H_2_ at 5 °C in order to verify if the CO methanation is possible using ultrasound. The gas chromatogram for CO methanation experiment is presented in Fig. 14 where FID channel shows the peak of methane confirming the formation of methane from CO. A methane yield of 0.4% was observed from 2% CO. Therefore, CO is an intermediate product in CO_2_ methanation process.

Mechanism 2: Ultrasound induced radical driven CO_2_ methanation

The H^•^ produced during ultrasonication (according to equations (8), (9) and (10)) react with CO_2_ to produce CO according to the equation (14) which then undergoes a series of radical reactions (Reaction 15 - Reaction 20) to produce CH_4_, C_2_H_4_ and C_2_H_6_.

(14) $$CO_{2}+H^{\cdot }\to CO_{2}H^{\cdot }\to CO+OH^{\cdot }$$

(15) $$\mathrm{CO}+4H^{\cdot }\to CH_{2}^{\cdot }+H_{2}O$$

(16) $$CH_{2}^{\cdot }+H^{\cdot }\to CH_{3}^{\cdot }$$

(17) $$CH_{3}^{\cdot }+H^{\cdot }\to CH_{4}$$

(18) $$CH_{2}^{\cdot }+CH_{2}^{\cdot }\to C_{2}H_{4}$$

(19) $$CH_{2}^{\cdot }+CH_{2}^{\cdot }+2H^{\cdot }\to C_{2}H_{6}$$

(20) $$CH_{3}^{\cdot }+CH_{2}^{\cdot }+OH^{\cdot }\to CH_{3}CH_{2}OH$$

## 5 Conclusions

In this study, we investigated the possibility to carry out the *Sabatier process* at ambient conditions in the absence of a catalyst, using power ultrasound only. It was found that when a small quantity of CO_2_ (less than3%) mixed with an inert gas is irradiated by power ultrasound, CO is formed, including a trace amount of methane confirming the occurrence of the *Sabatier process*. In this process, the reverse water gas shift reactions also occur at ambient conditions. However, when the inert gas is replaced by molecular hydrogen, a drastic improvement is achieved. In the presence of higher hydrogen concentrations, another major reaction called the *Fischer-Tropsch* process might take place producing higher carbon number-based hydrocarbons such as C_2_H_4_ and C_2_H_6_.

**Fig. 15:**
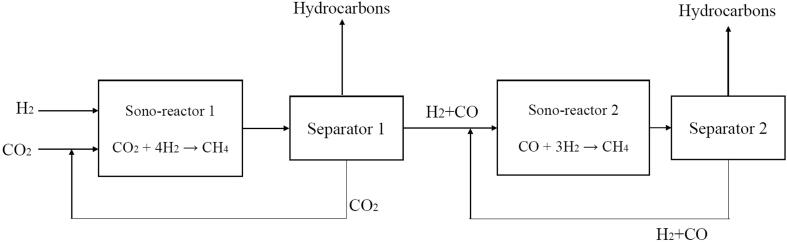
Conceptual design of a two-step process for CO_2_ reduction under ultrasonication.

Another improvement in the process has been achieved when 1.00 M NaCl or seawater is used as ultrasonicating media instead of pure water. It was found that the salt concentration in this range (0.40 M to 1.00 M) has a beneficial effect in the sonochemistry of gases involving CO_2_ and H_2_. NaCl tends to reduce the formation of H_2_O_2_ which is an oxidizing agent. A 1.00 M NaCl solution with a high hydrogen content (98%) in the gas mixture exhibits an excellent synergistic effect by creating a global reducing environment in the system facilitating the CO_2_ reduction process through this process*.* Under these conditions, around 5% total hydrocarbon yield was achieved. In addition to this, we have demonstrated that the CO_2_ content from synthetic industrial flue gas can also be converted into valuable hydrocarbons by diluting it using hydrogen. We have shown that, the salt content in the seawater has beneficial effects on the process where around 40% higher hydrocarbon yield was achieved. We have named this novel alternative method for the chemical CO_2_ reduction under ultrasonication as the “Islam-Pollet-Hihn process” (Fig. 15).

## CRediT authorship contribution statement

**Md Hujjatul Islam:** Conceptualization, Data curation, Formal analysis, Investigation, Methodology, Project administration, Software, Validation, Visualization, Writing - original draft. **Odne S. Burheim:** Funding acquisition, Conceptualization, Supervision, Investigation. **Jean-Yves Hihn:** Conceptualization, Data curation, Formal analysis, Investigation, Methodology, Project administration, Resources, Software, Supervision, Validation, Visualization, Writing - review & editing. **Bruno.G. Pollet:** Conceptualization, Data curation, Formal analysis, Funding acquisition, Investigation, Methodology, Project administration, Resources, Software, Supervision, Validation, Visualization, Writing - review & editing.

## Declaration of Competing Interest

The authors declare that they have no known competing financial interests or personal relationships that could have appeared to influence the work reported in this paper.
